# A potential strategy for bladder cancer treatment: inhibiting autophagy to enhance antitumor effects of Nectin-4-MMAE

**DOI:** 10.1038/s41419-024-06665-y

**Published:** 2024-04-25

**Authors:** Yichen Wang, Yanyang Nan, Chunguang Ma, Xiaolin Lu, Qian Wang, Xiting Huang, Wenjing Xue, Jiajun Fan, Dianwen Ju, Dingwei Ye, Xuyao Zhang

**Affiliations:** 1 Department of Urology, Fudan University Shanghai Cancer Center, Department of Oncology, Shanghai Medical College, Fudan University, Shanghai, 200032 China; 2 Department of Biological Medicines & Shanghai Engineering Research Center of Immunotherapeutic, Fudan University School of Pharmacy, Shanghai, 201203 China

**Keywords:** Cancer therapeutic resistance, Bladder cancer

## Abstract

Research and development on Nectin-4 antibody-drug conjugates (ADC) have been greatly accelerated since the approval of enfortumab vedotin to treat uroepithelial cancer. During the course of this study, we identified that autophagy serves as a cytoprotective mechanism during Nectin-4-MMAE treatment and proposed a strategy to enhance the antitumor effects of Nectin-4-MMAE in bladder cancer. Nectin-4-MMAE rapidly internalized into bladder cancer cells in 30 minutes and released MMAE, inducing the onset of caspase-mediated apoptosis and leading to the inhibition of tumor cell growth. Transcriptomics showed significant alterations in autophagy-associated genes in bladder cancer cells treated with Nectin-4-MMAE, which suggested autophagy was activated by Nectin-4-MMAE. Furthermore, autophagy activation was characterized by ultrastructural analysis of autophagosome accumulation, immunofluorescence of autophagic flux, and immunoblotting autophagy marker proteins SQSTM1 and LC3 I/II. Importantly, inhibiting autophagy by LY294002 and chloroquine significantly enhances the cytotoxicity effects of Nectin-4-MMAE in bladder cancer cells. Additionally, we detected the participation of the AKT/mTOR signaling cascade in the induction of autophagy by Nectin-4-MMAE. The combination of Nectin-4-MMAE and an autophagy inhibitor demonstrated enhanced antitumor effects in the HT1376 xenograft tumor model. After receiving a single dose of Nectin-4-MMAE, the group that received the combination treatment showed a significant decrease in tumor size compared to the group that received only one type of treatment. Notably, one mouse in the combination treatment group achieved complete remission of the tumor. The combination group exhibited a notable rise in apoptosis and necrosis, as indicated by H&E staining and immunohistochemistry (cleaved caspase-3, ki67). These findings demonstrated the cytoprotective role of autophagy during Nectin-4-MMAE treatment and highlighted the potential of combining Nectin-4-MMAE with autophagy inhibitors for bladder cancer treatment.

## Introduction

Antibody-drug conjugate (ADC) has made significant advancements in recent years, particularly in the context of treating Her-2-positive solid tumors [1]. As a type I transmembrane protein that is aberrantly expressed in several cancer types, nectin-4 (also known as PVRL4) has emerged as a highly promising target for ADC research and development [2, 3]. Enfortumab vedotin (EV) is the first FDA-approved nectin-4 ADC for metastatic urothelial carcinoma. However, in EV-301, a global, open-label, phase III trial of EV, ~30% of patients failed to receive benefits from EV [4]. Meanwhile, primary and secondary resistance to ADC seems to be inevitable [5–7]. Downregulation of antigens [8] and impaired drug transport [9] were reported in various ADC. Hence, mining the underlying mechanisms and maximizing the potential of ADCs to benefit more patients remains an urgent issue to be addressed.

Autophagy, specifically macroautophagy, is a conserved cellular process involved in the degradation of damaged organelles or proteins through the formation of double-membrane autophagosomes [10]. These autophagosomes subsequently fuse with lysosomes to form autophagolysosomes, promoting degradation of their contents [11]. Cells can endure and survive in situations of limited nutrients or stress via autophagy [12]. Several research studies indicated that autophagy may act as a protective mechanism for cancer cells in the initial phases in reaction to stress caused by medication therapy [13–15]. Conversely, excessive autophagy can lead to autophagic cell death [16], making autophagy a double-edged sword [17]. In the limited studies on ADC therapy and autophagy, the variable role of autophagy in ADC treatment is still unclear [18, 19]. Therefore, the questions of whether Nectin-4 ADC induces autophagy and what is the role of autophagy in Nectin-4 ADC treatment are of great interest for improving the therapeutic efficacy of Nectin-4 ADC.

In this study, we utilized Nectin-4-MMAE (DAR4), a Nectin-4 ADC in Phase I/II trials, to elucidate these questions. Monomethyl auristatin E (MMAE) is a microtubule inhibitor with high cytotoxicity and is widely used in the payloads of ADC. In vitro and in vivo settings, our findings indicate that Nectin-4-MMAE exhibits substantial cytotoxicity on human bladder cancer cells. More importantly, we found that autophagy was triggered by Nectin-4-MMAE. Notably, combining autophagy inhibitors with Nectin-4-MMAE further enhances the antitumor efficacy of Nectin-4-MMAE. Our results reveal that inhibiting autophagy strengthens the antitumor effects of Nectin-4-MMAE, underscoring the potential of a combined therapeutic approach utilizing Nectin-4-MMAE and autophagy inhibitors for more effective bladder cancer treatment.

## Materials and methods

### Reagent and antibody

Nectin-4-MMAE is a gift from Mabwell Bioscience CO., LTD. Shanghai China. Autophagy modulators: LY294002 (Medchemexpress, HY-10108, Shanghai China); Chloroquine (Sigma-Aldrich, C6628, Darmstadt Germany); Rapamycin (Sangon Biotech, A606203, Shanghai China). Cell viability assay: MTT (Beyotime, C0009S, Shanghai, China). ADC labeling: AlexaFlour 488 protein label kit (ThermoFisher, A10235, MA US). Cell apoptosis assay: cell apoptosis detection kit (Meilunbio MA0220-1, Dalian China). Autophagy assay: Cyto-ID autophagy detection kit (Enzo Life Sciences, ENZO-51031-K200, NY US); Lyso-Tracker-DND 99 (Invitrogen, L7528, CA US). Antibodies: Primary antibody: anti-GAPDH, #2118; anti-LC3 I/II, #3868; anti-SQSTM1, #8025; anti-caspase 3, #9662; anti-PARP, #9542; anti-phospho-mTOR (Ser2448), #2971; anti-mTOR, #2983; anti-phospho-AKT (Ser473), #4060; anti-phospho-p70s6 Kinase (Ser371), #9208; anti-p70s6 Kinase, #2708; anti-phospho-4EBP1 (Thr45), #2971; anti-4EBP1, #9644, anti-PI3 Kinase, #4257; anti-phospho-PI3 Kinase, #4228 (Cell Signaling Technology, MA US). Anti-PDK-1, AF7707 (Beyotime Biotechnology, Shanghai, China). Anti-PTEN, A19104 (Abclonal, Wuhan China). Secondary antibody: HRP-conjugated anti-rabbit secondary antibody, #7074; anti-mouse IgG secondary antibody, #7076 (Cell Signaling Technology, MA US).

### Cell culture

Human bladder cancer cells HT1376, SW780 (nectin-4 positive) and 5637, T24 (nectin-4 negative) were procured from Nanjing Cobioer Bioscience CO., LTD, Nanjing, China and Type Culture Collection of the Chinese Academy of Sciences, Shanghai, China respectively. HT1376 cells were cultured in MEM medium with 1% Nonessential Amino Acids. SW780 and, 5637, T24 were cultured in DMEM and RPMI-1640 medium, respectively. All mediums contained 10% fetal bovine serum, penicillin (100 U/ml), and streptomycin (100 μg/mL). All cell cultures were maintained in a 5% CO_2_ humidified atmosphere at 37 °C. A routine test for mycoplasma was performed to ensure all cell lines were mycoplasma-free.

### Cell viability assay

Cell viability of bladder cancer cells was assessed using an MTT assay. Bladder cancer cells were incubated with Nectin-4-MMAE with/without autophagy inhibitors. Following the indicated co-incubation period, 0.5 mg/mL MTT was pipetted into the cells for 4 hours at 37 °C. The formazan products were dissolved by DMSO, and the absorbance was measured at 490 nm. Cell viability (%)

$$=\frac{{\text{Mean}}\, {\text{absorbance}}\;({\text{test}}\,{\text{group}})}{{\text{Mean}}\,{\text{ absorbance}}\;({\text{vehicle}}\,{\text{group}})}\times 100 \% \left({\it{n}}=5\right).$$

### RNA-seq assay

The TRIzol method was utilized for the extraction of total RNA, and DNase 1 was employed to eliminate genomic DNA. To ensure the RNA sample’s quality, they were assessed using the 2100 bioanalyser and the ND-2000. The examination aimed to ensure that OD260/280 = 1.8 to 2.2, OD260/230 ratio is above 2.0, RIN is higher than 6.5, 28 S:18 S ratio is above 1.0, and the quantity is <1 μg. The TruSeqTM RNA Sample Preparation Kit was utilized to construct RNA libraries, and the Illumina HiSeq ten/NovaSeq 6000 sequencing platform was employed for high-throughput sequencing. Genes related to autophagy were gained from the Human Autophagy Database (HADb). The GSEA software was utilized to conduct Gene set enrichment analysis (GSEA) (*n* = 3).

### Transmission electron microscopy

HT1376 and SW780 cells were incubated with or without Nectin-4 ADC for the specified duration, harvested, and prepared following established procedures. Samples were visualized using a HITACHI HT7800 transmission electron microscopy (TEM).

### Confocal microscopy

HT1376 and SW780 cells were treated with Nectin-4 ADC for the specified duration. Subsequently, Cyto-ID was employed to detect autophagosomes. Rapamycin (50 nM) incubated cells acted as the positive control. Cells treated as described above were observed using Carl Zeiss LSM710 (Carl Zeiss, Germany).

### Apoptosis analysis

The Annexin V-FITC/PI Apoptosis Detection Kit was employed following the manufacturer’s instructions to evaluate apoptosis in HT1376 and SW780 cells. Analysis was conducted using a FACS Calibur flow cytometer (Becton-Dickinson, Fullerton, CA) (*n* = 3).

### Western blot analysis

Cell lines were lysed with RIPA lysis buffer (0 °C, 30 min). The cell lysates were separated by centrifugation (12,000 rpm, 15 min). Protein concentrations were determined by BCA. Primary antibodies were incubated overnight at 4 °C, secondary antibodies were incubated for 2 hours at room temperature. The proteins were then detected by chemiluminescence. The gray value was measured by Image J software (*n* = 3).

### Xenograft model

1 × 10^7^ HT1376 cells suspended in PBS were injected in mice (BALB/c nude, male, 6 weeks, purchased from GemPharmatech) subcutaneously. Tumor-bearing mice were randomly allocated into 4 groups, each group contained 5 mice. The sample size of mice was determined based on literature documentation of similar well-characterized experiments. Nectin-4 ADC was administered as a single intravenous dose, while LY294002 was given via intraperitoneal injection twice a week. The animal studies were blinded, and all animal procedures were conducted in compliance with protocols approved by the Animal Ethical Committee of the School of Pharmacy, Fudan University.

### Statistical analysis

Data analysis was performed using GraphPad Prism 8 (GraphPad Software Inc., San Diego, CA). All experiments were analyzed in a blinded manner and repeated at least three times. The results were expressed as means ± standard deviation. Normal distribution was tested by the Shapiro–Wilk test. Comparisons were carried out using Student’s *t* test (two-tailed) and Mann–Whitney test and a *p* value <0.05 was considered statistically significant. Sample sizes were determined by power analysis with a power of 80%, *α* = 0.05. No data or animals were excluded from the analyses. Adobe Photoshop and Illustrator were employed for figure preparation, and the graphical illustration was drawn by Figdraw.

## Results

### Nectin-4-MMAE induced apoptosis and elicited potent cytotoxicity in nectin-4 positive bladder cancer cells

**Fig. 1:**
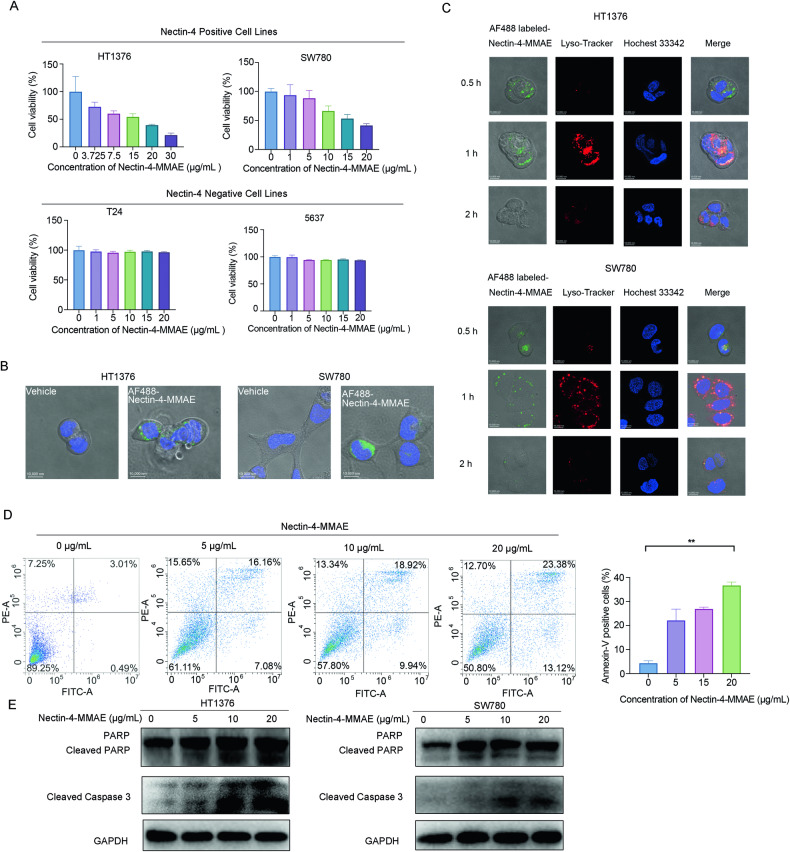
Nectin-4-MMAE elicited potent cytotoxicity and induced apoptosis in nectin-4-positive bladder cancer cells. **A** Nectin-4 positive and negative cell lines were treated with indicated concentrations of Nectin-4-MMAE for 72 hours, and cell viability was detected by MTT. **B** H1376 and SW780 cells were incubated with AlexaFlour 488-labeled Nectin-4-MMAE for 30–60 minutes in 37 °C, cells were then analyzed by confocal microscopy. **C** To track the intracellular routes of Nectin-4-MMAE, HT1376, and SW780 cells were stained with Lysotracker, and then incubated with AlexaFlour 488-labeled Nectin-4-MMAE at 37 °C for the indicated time, then cells were analyzed by confocal microscopy. Blue fluorescence, Hoechst 33342 staining; red fluorescence, lysosome; green fluorescence, Nectin-4-MMAE. **D** Flow cytometric analyses of Annexin V-FITC were employed to detect apoptosis induced by Nectin-4-MMAE in HT1376 cells. **P < 0.01. **E** The protein expression of PARP, cleaved-PARP, and cleaved-Caspase-3 was assessed by western blot.

Initially, our study focused on investigating the specific cytotoxicity of Nectin-4-MMAE towards nectin-4 positive cells along with its internalization process. Two nectin-4 positive bladder cancer cells, HT1376 and SW780, were used to assess the cytotoxic effects of Nectin-4 ADC. Nectin-4 negative bladder cancer cells 5637 and T24 were designated as negative controls. The results demonstrated a significant and dose-dependent decrease in cell viability for both HT1376 and SW780 after 72 hours of Nectin-4-MMAE treatment. Notably, at a concentration of 30 μg/mL, Nectin-4-MMAE achieved an inhibition rate of up to 80% for HT1376 (Fig. 1A). Conversely, Nectin-4-MMAE exhibited minimal cytotoxicity towards 5637 and T24, even at high concentrations (Fig. 1A).

The internalization and subsequent lysosomal degradation of ADC drugs are critical processes for their antitumor effects. To investigate whether Nectin-4-MMAE could internalize into cells, we employed AlexaFluor 488-labeled Nectin-4-MMAE (10 μg/mL) and incubated it with HT1376 and SW780 cells. Using laser confocal microscopy, we observed green fluorescence inside the cells 30-60 minutes after administration, indicating efficient binding to cell surface receptors and internalization (Fig. 1B). Additionally, we stained cellular lysosomes using Lysotracker and observed significant activation (red fluorescence) around 60 mins after administration, with high co-localization between Nectin-4-MMAE (green fluorescence) and lysosomes. This suggests the successful transport of Nectin-4-MMAE to lysosomes after cellular internalization. After 2 hours, the green fluorescence gradually diminished, possibly due to the degradation of antibody molecules within Nectin-4-MMAE (Fig. 1C). These findings collectively demonstrate that Nectin-4-MMAE efficiently binds to cell surface receptors, enters cells, and undergoes degradation in lysosomes, enabling payload release for tumor cell killing.

Subsequently, we delved into the mechanism of cell killing by Nectin-4-MMAE, which utilizes MMAE as its payload, a microtubule protein inhibitor that disrupts cell mitosis, leading to various forms of cell death. We initiated investigations into apoptosis by employing Annexin V/PI double staining. The results revealed a dose-dependent increase in the proportion of cells undergoing early apoptosis (Annexin V+ PI−) or late apoptosis (Annexin V+ PI+) with increasing Nectin-4-MMAE dose. This observation suggests that Nectin-4-MMAE induces apoptosis in Nectin-4-positive cells (Fig. 1D). Furthermore, we examined classical indicators of apoptosis, such as caspase-3 and PARP, and observed a significant increase in the cleavage of caspase-3 and PARP. These data provide evidence that Nectin-4-MMAE induced cell apoptosis and potent cytotoxicity in nectin-4 positive bladder cancer cells (Fig. 1E).

### Nectin-4-MMAE induced increased transcription of autophagy positively regulated genes

**Fig. 2:**
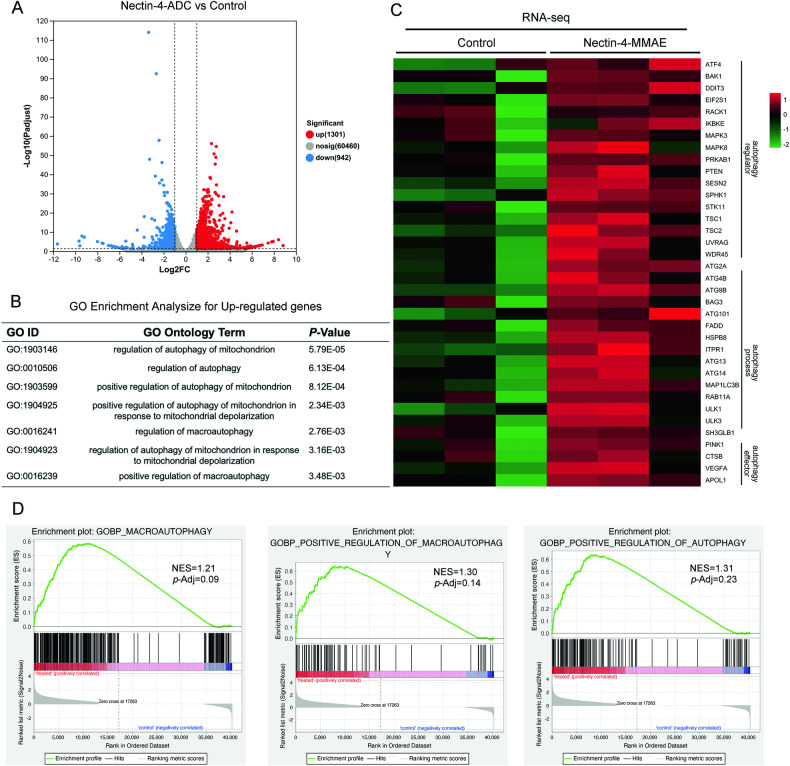
Nectin-4-MMAE activated autophagy gene network in nectin-4 positive bladder cancer cells. HT1376 cells were incubated with Nectin-4-MMAE (10 μg/mL) for 48 hours; then the total RNA was extracted for mRNA-seq. **A** Volcano plots showed the adjusted *p* value for genes differentially expressed between control HT1376 cells and HT1376 cells treated with Nectin-4-MMAE. **B** Gene Ontology (GO) analysis of the autophagy-related genes that showed in upregulated genes. **C** Heatmap of changes in mRNA levels of autophagy-related genes (*n* = 3). **D** GSEA analysis showed the gene profiles of HT1376 incubated with Nectin-4-MMAE enriched in macroautophagy, positive regulation of macroautophagy and positive regulation of autophagy. NES and *p*-adj were indicated.

To gain a more comprehensive understanding of how tumor cells respond to Nectin-4-MMAE treatment, we conducted transcriptome RNA sequencing to assess changes in gene expression. After 48 hours of treatment with 10 μg/mL Nectin-4-MMAE, a significant amount of genes in HT1376 cells were found to be either upregulated or down-regulated, as indicated by the resulting volcano plot. In particular, there was an increase in the expression of 1301 genes. Whereas 942 genes showed a decrease in expression (Fig. 2A). We subsequently performed an analysis of Gene Ontology (GO) enrichment on the genes that were up-regulated, which showed notable enrichment in various GO categories associated with autophagy (Fig. 2B). Further analysis focused on key autophagy-related genes, including effectors of autophagy, genes involved in the autophagy process, lysosome-related genes, and genes that regulate autophagy. The results demonstrated significant upregulation in the expression of these genes after Nectin-4-MMAE treatment (Fig. 2C). Moreover, GSEA indicated that Nectin-4-MMAE treatment led to the enrichment of several pathways that are positively regulated by autophagy (Fig. 2D). Through this comprehensive transcriptome analysis, we have identified evidence suggesting that Nectin-4-MMAE treatment may activate autophagy. Our next step involves validating the occurrence of autophagy in tumor cells following Nectin-4-MMAE treatment at the cellular level.

### Nectin-4-MMAE induced the formation and accumulation of autophagosomes and autophagy flux

**Fig. 3:**
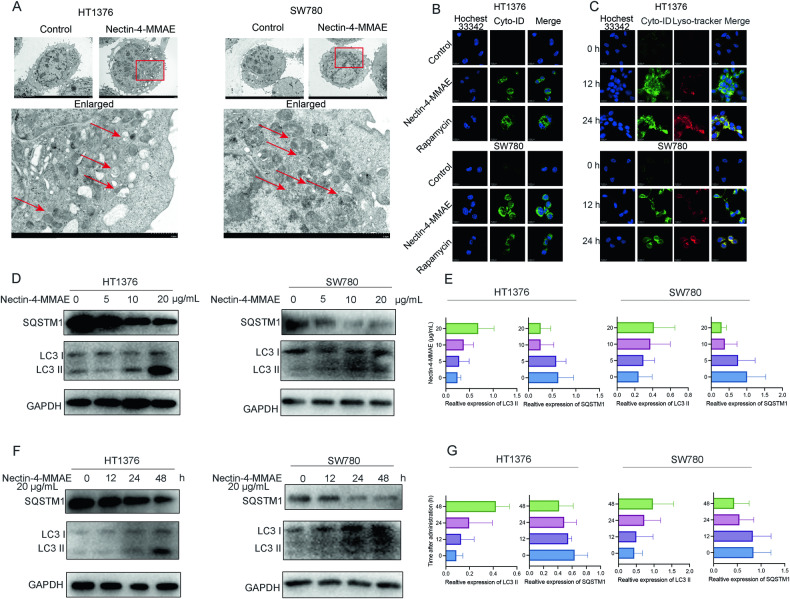
Nectin-4-MMAE induced the formation and accumulation of autophagosomes and autophagy flux. **A** TEM analysis of autophagosome (red arrowhead) in HT1376 and SW780 cells incubated with Nectin-4-MMAE. **B** HT1376 and SW780 cells were treated with Nectin-4-MMAE and autophagosomes stained with Cyto-ID were examined by confocal microscopy, cells treated with rapamycin were employed as the positive control. **C** To detect autophagosome-lysosome fusion and autophagy flux, Cyto-ID and Lysotracker were utilized to stain autophagosomes and lysosomes, respectively. After incubation with Nectin-4-MMAE for the indicated time, HT1376 and SW780 were analyzed under confocal microscopy. **D** The protein expression of LC3 I/II and SQSTM1 in HT1376 and SW780 treated by the indicated concentration of Nectin-4-MMAE were assessed by western blot. **E** The quantification of **D** using Image J software. **F** The protein expression of LC3 I/II and SQSTM1 in HT1376 and SW780 treated by Nectin-4-MMAE (20 μg/mL) for indicated durations were assessed by western blot. **G** The quantification of **F** using Image J software.

The gold standard for confirming autophagy is the observation of autophagosomes using TEM. In our study, we initially utilized TEM to examine cells treated with Nectin-4-MMAE for 12 hours. TEM images revealed a notable increase in vesicles exhibiting double-layer membrane structures in ΗΤ1376 and SW780 cells following treatment with 10 μg/mL of Nectin-4-MMAE, as compared to the control group. This observation unequivocally demonstrates that Nectin-4-MMAE induces the accumulation of autophagosomes within cells (Fig. 3A). We further employed confocal microscopy to examine Nectin-4-MMAE-treated cells after staining with the autophagosome dye Cyto-ID. This analysis revealed a substantial increase in green fluorescence intensity after 12 hours of Nectin-4-MMAE treatment, comparable to the positive control rapamycin. This finding strongly suggests the production and accumulation of autophagosomes in Nectin-4-MMAE-treated cells (Fig. 3B). To delve deeper, we assessed the autophagic flux, specifically the conversion of autophagosomes to autophagolysosomes and their subsequent degradation. In HT1376 and SW780 cells treated with 10 μg/mL of Nectin-4-MMAE for 12 hours, we observed the accumulation of autophagosomes (green fluorescence) and their co-localization with lysosomes (red fluorescence). After 24 hours of treatment, we witnessed the formation and degradation of autophagolysosomes (yellow fluorescence), providing compelling evidence for the complete occurrence of autophagy induced by Nectin-4-MMAE (Fig. 3C). Furthermore, we evaluated changes in the expression of autophagy marker proteins LC3 I/II and SQSTM1. Immunoblot results demonstrated a dose-dependent and time-dependent increase in the expression of LC3-II, contrasting with a dose and time-dependent decrease in the expression of SQSTM1 (Fig. 3D–G). These results reinforce the assertion that Nectin-4-MMAE effectively induces autophagy.

**Fig. 4:**
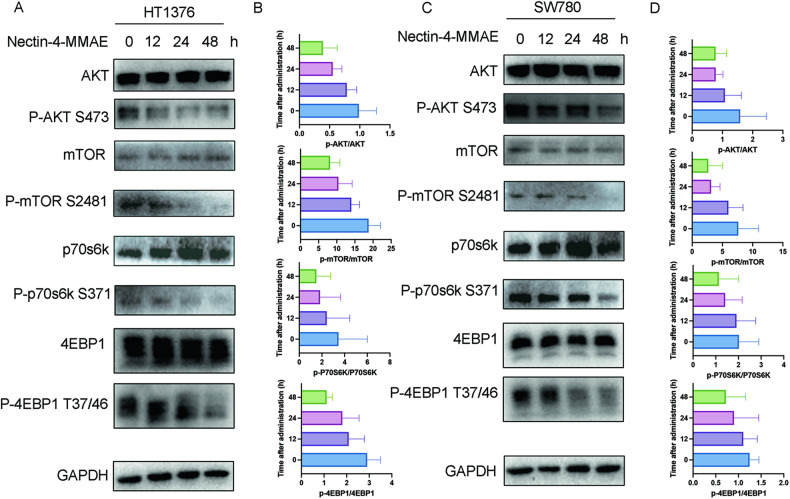
Akt/mTOR pathway was inactivated in Nectin-4-MMAE-treated nectin-4 positive bladder cancer cells. **A**, **C** Western blot analysis for the AKT/mTOR pathway showed the total protein levels and phosphorylation levels of Akt, mTOR, P70S6K, 4EBP1 in HT1376 and SW780 treated with Nectin-4-MMAE for the indicated time. **B**, **D** The quantification of p-Akt, p-mTOR, p-P70S6K, p-4EBP1 normalized by total Akt, mTOR, P70S6, and 4EBP1 in **A**, **C**.

Then, we investigated the mechanism behind Nectin-4-MMAE-induced autophagy. Our findings revealed a time-dependent decrease in the phosphorylation levels of mTOR in HT1376 and SW780 cells following Nectin-4-MMAE treatment. Moreover, phosphorylation levels of Akt, an upstream regulator of mTOR, exhibited significant reductions. Remarkably, two downstream components of the Akt/mTOR pathway, p70S6k, and 4EBP1, exhibited significantly reduced phosphorylation levels (Fig. 4). To further investigate the molecular mechanism of Akt/mTOR inactivation, we examined the expression levels of PI3K, PDK-1 and PTEN, which are regulatory molecules upstream of Akt/mTOR, and PI3K/PDK-1 can activate the Akt/mTOR signaling pathway whereas PTEN reverses this process. The results showed that the phosphorylation level of PI3K was significantly reduced while the expression of PDK-1 was significantly decreased (Fig. S1). These results suggest that Nectin-4-MMAE may induce autophagy by inhibiting the Akt/mTOR signaling pathway.

Collectively, the aforementioned findings, in alignment with the results of biosignature analysis, affirm that Nectin-4-MMAE not only triggers the formation and accumulation of autophagosomes but also instigates the complete progression of autophagic flux via the inactivation of Akt/mTOR signaling pathway.

### Autophagy inhibition enhanced apoptosis and cytotoxicity induced by Nectin-4-MMAE

**Fig. 5:**
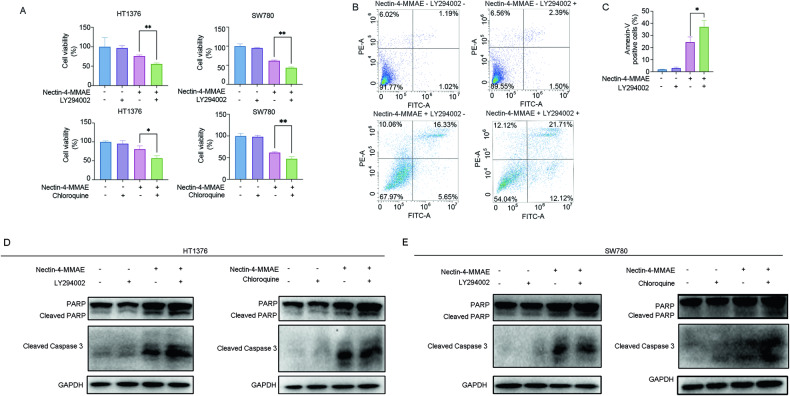
Autophagy inhibition enhanced apoptosis and cytotoxicity induced by Nectin-4-MMAE. **A** HT1376 and SW780 cells incubated with Nectin-4-MMAE and/or autophagy inhibitor (LY294002 and chloroquine), cell viability was detected by MTT. *P < 0.05, **P < 0.01. **B**, **C** Flow cytometric analysis of apoptosis. HT1376 cells were treated with Nectin-4-MMAE or LY294002 or the combination. *P < 0.05. **D**, **E** the protein expression of PARP, cleaved-PARP, and cleaved-Caspase-3 in HT1376 and SW780 were assessed by western blot.

As previously discussed, the role of autophagy in cancer therapy often presents a dual nature. To decipher the precise role of autophagy induced by Nectin-4 ADC in its therapeutic process, we employed autophagy inhibitors, LY294002 (3 μM) and chloroquine (5 μM), in combination with Nectin-4-MMAE to treat HT1376 and SW780. The results unequivocally demonstrated that the combination-treated groups exhibited significantly lower cell viability compared to the monotherapy groups (Fig. 5A). Flow cytometry analysis revealed a substantially higher proportion of apoptotic cells in the co-treated groups compared to the Nectin-4-MMAE-alone groups (Fig. 5B, C). Further examination of apoptosis-related proteins showed that the expression levels of cleaved-caspase 3 and cleaved-PARP were significantly elevated in the combination treatment groups compared to the Nectin-4-MMAE-alone treatment groups (Fig. 5D, E). These results collectively suggest that autophagy induced by Nectin-4-MMAE plays a protective role for tumor cells, and inhibiting autophagy can enhance the cytotoxic and apoptosis-inducing potential of Nectin-4-MMAE, thereby augmenting its therapeutic efficacy.

### Inhibition of autophagy enhanced the antitumor effects of Nectin-4-MMAE in xenograft tumor models

**Fig. 6:**
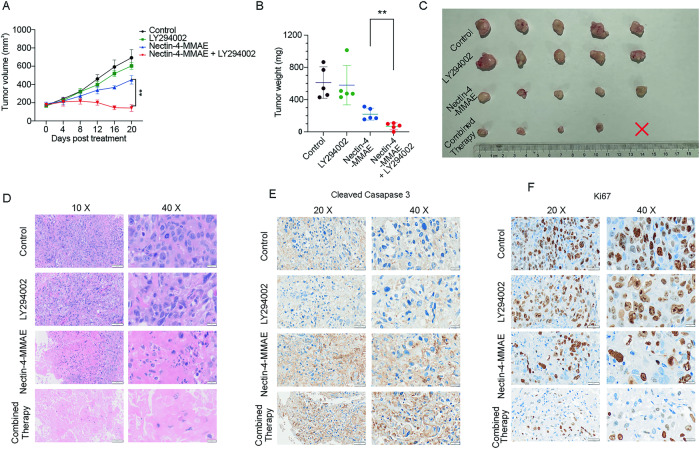
Inhibition of autophagy enhanced the antitumor effects of Nectin-4-MMAE in xenograft tumor models. Antitumor effect of Nectin-4-MMAE (3 mg/kg, single dose) in combination with autophagy inhibitor LY294002 (25 mg/kg, twice a week) in HT1376 xenograft. (*n* = 5 mice). **A** Tumor growth curve. Black line, Control; green line, LY294002; blue line, Nectin-4-MMAE; red line, combination therapy. **P < 0.01. **B** Tumor weight. Black dots, Control; green dots, LY294002; blue dots, Nectin-4-MMAE; red dots, combination therapy. **P < 0.01. **C** Image of tumor tissue. The red cross indicates tumor-free. **D**–**F** Hematoxylin and eosin (HE) and immunohistochemical staining for cleaved-caspase 3 and ki67 of tumor tissues from the indicated treatment groups.

To corroborate the conclusions drawn from in vitro experiments, we conducted in vivo studies. Specifically, we established HT1376 xenograft tumor models to investigate the impact of autophagy inhibition on the antitumor effect of Nectin-4-MMAE. Results indicated that tumors in the Nectin-4-MMAE and LY294002 combination treatment group began to shrink from day 8 post-treatment, with this trend continuing until the experiment’s conclusion. Conversely, this phenomenon was not observed in the other groups (Fig. 6A). Notably, tumor weight in the combination treatment group (69 ± 43.13 mg) was significantly lower than that in the Nectin-4-MMAE-alone treatment group (219.8 ± 73.75 mg). In contrast, no significant differences were noted in tumor weights between the autophagy inhibitor-alone treatment group and the control group (580.2 ± 244.0 mg and 612.4 ± 194.6 mg, respectively) (Fig. 6B). Remarkably, one individual in the combination treatment group achieved complete tumor remission (Fig. 6C). Histological examination via H&E staining revealed lower tumor cell density and an increased presence of necrotic cells in tumor tissues of the combination therapy group (Fig. 6D). Immunohistochemical results for cleaved-caspase 3 and ki67 demonstrated higher cleaved-caspase 3 expression in the combination therapy group compared to the Nectin-4-MMAE-alone group, along with lower ki67 expression in the combination therapy group. In conclusion, these results provide further evidence that inhibition of autophagy significantly enhances the ability of Nectin-4-MMAE to induce apoptosis and cytotoxicity in tumors, thereby amplifying the antitumor efficacy of Nectin-4-MMAE.

## Discussion

Despite the remarkable strides achieved by ADCs in combating solid tumors, their widespread adoption as a first-line treatment option is hindered by a low response rate and the continuous emergence of resistance. In the EV-301 trial, ~30% of patients did not derive benefit from Nectin-4 ADC treatment [4]. Similar challenges persist across various ADCs. For instance, in the Phase II/III clinical trial, T-DM1, the inaugural HER-2 ADC approved, did not exhibit a more robust impact than taxane in Her-2-positive gastric cancer [20]. These findings have spurred our investigation into the underlying mechanisms, aiming to surmount these obstacles and elevate the efficacy of ADCs.

Autophagy, a complex cellular process, assumes a pivotal role in tumor treatment. However, conflicting outcomes from various studies have led to disparate conclusions regarding the impact of autophagy. For instance, Zhang et al. reported that CD47 blocking triggered autophagy, enhancing macrophage phagocytosis and antitumor efficacy [21], while different research on T-DM1 suggested a cytoprotective role of autophagy, shielding tumor cells from T-DM1-induced death [18]. Nevertheless, excessive autophagy can lead to autophagic cell death in specific contexts [19, 22, 23]. In our investigation, we present novel evidence elucidating the induction of autophagy in nectin-4-positive tumor cells following Nectin-4 ADC exposure. Our mRNA-seq analysis unveiled a significant upregulation of autophagy-related genes in the bladder cancer cells treated with Nectin-4-MMAE, spanning from the regulatory to the executive stages of autophagy. This induction of autophagy was further validated by the observation of increased autophagosome accumulation, the occurrence of autophagic flux, and elevated expression of LC3-II induced by Nectin-4-MMAE. Our findings highlight the cytoprotective role of autophagy during Nectin-4-MMAE treatment. Inhibiting autophagy not only enhances Nectin-4-MMAE cytotoxicity but also promotes Nectin-4-MMAE-induced apoptosis. Extensive research has demonstrated the crosstalk between autophagy and apoptosis. Autophagy modulation influences apoptosis induction through multiple pathways [24–26]; attenuation of apoptosis due to autophagy has also been reported in multiple treatments, including immunotherapy [21], chemotherapy [27], and radiotherapy [28]. Consistent with these studies, inhibition of autophagy using LY294002 or chloroquine resulted in increased cleavage of caspase-3 and PARP, elevated Annexin V-positive cells, increased cleaved-caspase-3, and reduced Ki67 in xenograft tissues. Additionally, we observed inactivation of the Akt/mTOR pathway by Nectin-4-MMAE, likely mediating autophagy induction. The Akt/mTOR pathway, crucial in tumor cell proliferation, metabolism, and survival [29], exhibits a dual role: its inactivation suppresses tumor growth but also triggers autophagy. Our comprehensive findings delineate that Nectin-4-MMAE-induced autophagy impairs apoptosis and subsequently diminishes the antitumor efficacy of Nectin-4-MMAE.

In tandem with the enrichment of autophagy-associated pathways in our mRNA sequencing outcomes, we identified significant enrichment of pathways pertinent to mitochondrial autophagy, such as “positive regulation of autophagy of mitochondrion” and “positive regulation of autophagy of mitochondrion in response to mitochondrial depolarization”. Mitochondrial autophagy, a form of selective autophagy incited by oxidative stress and mitochondrial impairment, is notably triggered by mitochondrial depolarization, a common occurrence during apoptosis [30, 31]. This suggests that mitochondrial autophagy might represent a resistance mechanism against apoptosis in tumor cells. Our data indicates that the resistance of tumor cells to Nectin-4-MMAE stems from a multifaceted interplay involving various types of autophagy. While it is conceivable to counteract their effects collectively through a unified strategy (e.g., employing a PI3K inhibitor), a deeper exploration using more specific methodologies is imperative to discern the predominant contributions of each type of autophagy.

Numerous endeavors have been dedicated to enhancing the efficacy of ADCs. Beyond the quest for more precise targets and the refinement of the properties of antibodies and linkers [32], combination therapy stands out as a pivotal strategy [33, 34]. Notably, the utilization of tyrosine kinase inhibitors (TKIs) and CDK4/6 inhibitors has been instrumental in bolstering the effectiveness of ADCs [35, 36]. Moreover, the conjunction of ADCs with immune checkpoint inhibitors has displayed promising therapeutic outcomes in various clinical trials [37, 38]. In our study, we introduce a novel therapeutic approach by proposing the combination of Nectin-4-MMAE and autophagy inhibitors for bladder cancer treatment. Within the xenograft model, a pronounced reduction in tumor size was evident in the group subjected to combination therapy. Notably, one mouse within the combination treatment cohort exhibited a complete disappearance of the tumor. Immunohistochemical analysis further substantiated these findings, revealing an increased occurrence of apoptosis and necrosis in the tissues of the combination therapy group, consistent with our in vitro experimental outcomes. These collective results strongly indicate that employing autophagy inhibitors can nullify the protective effects of autophagy during Nectin-4-MMAE treatment, thereby augmenting the antitumor efficacy of Nectin-4-MMAE. This strategy holds promise as a potential clinical therapeutic intervention.

**Fig. 7:**
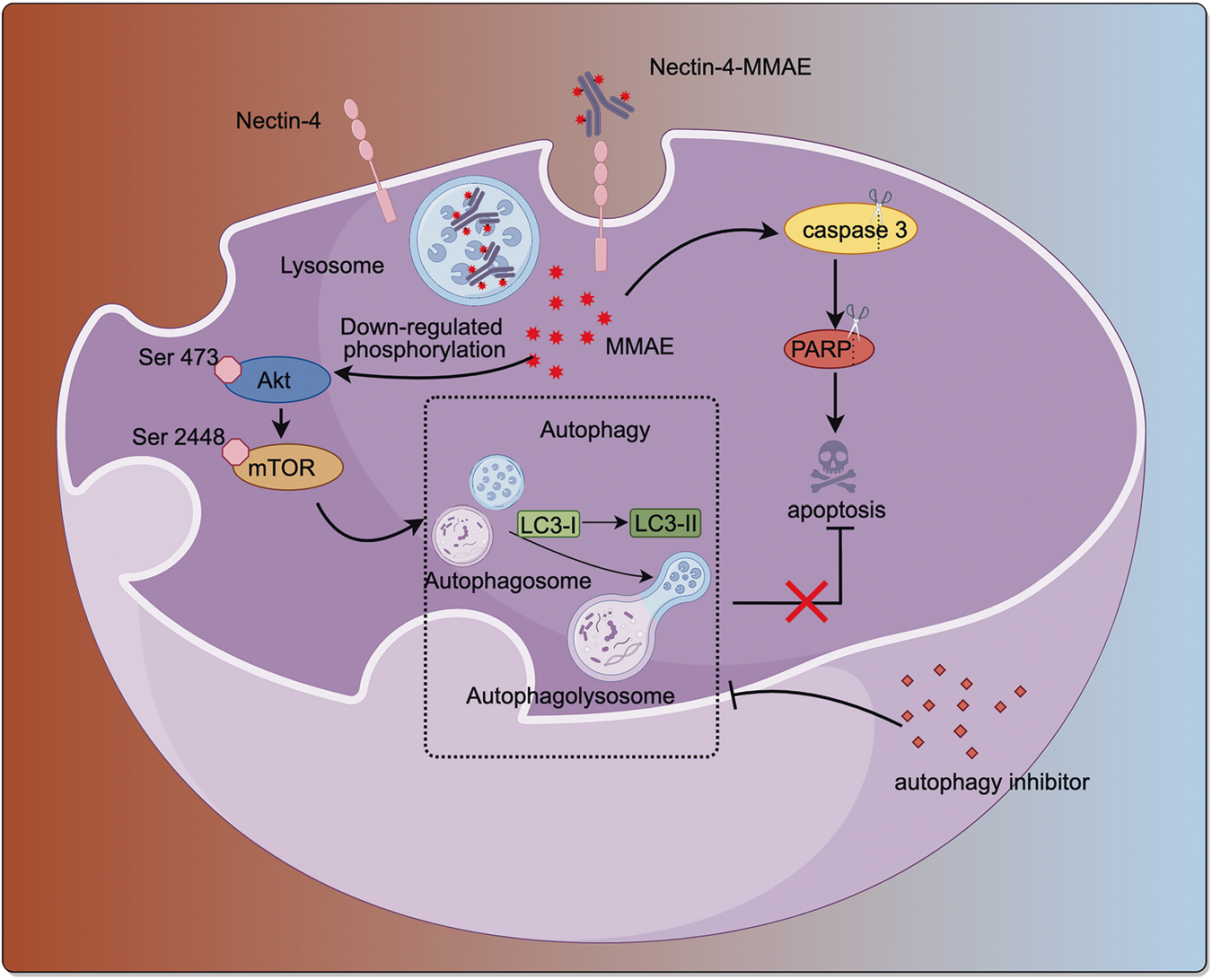
Autophagy Inhibition Enhances the anti-tumor Effects of Nectin-4-MMAE. A graphical illustration of how autophagy protects tumor cells in the presence of Nectin-4-MMAE and how blocking autophagy enhances the antitumor effects of Nectin-4-MMAE.

In summary, Nectin-4-MMAE showed antitumor effect via caspase-dependent apoptosis in bladder cancer cells. During the process, the induction of autophagy by Nectin-4-MMAE was observed in both HT1376 and SW780 cells. Our study further underscored the cytoprotective role of autophagy during Nectin-4-MMAE treatment (Fig. 7). Inhibition of autophagy substantially enhances apoptosis and cytotoxicity induced by Nectin-4-MMAE, and potentiates the antitumor efficacy of Nectin-4-MMAE in vivo. Therefore, the proposed approach of combining Nectin-4-MMAE with autophagy inhibitors presents a promising avenue for bladder cancer treatment, underpinned by robust experimental validation and theoretical rationale.

### Supplementary information

Figure S1.docx

Supplemental material (western blot)

## Data Availability

The published article includes all data sets generated/analyzed for this study.
