# Supplementary material for: A potential strategy for bladder cancer treatment: inhibiting autophagy to enhance antitumor effects of Nectin-4-MMAE
Source: Cell Death Dis. 2024 Apr 25;15(4):293. doi: 10.1038/s41419-024-06665-y (PMC11045801; doi:10.1038/s41419-024-06665-y)
Supplement: Supplementary file 1 — Figure S1.docx [file 41419_2024_6665_MOESM1_ESM.docx]

**A Potential Strategy for Bladder Cancer Treatment: Inhibiting Autophagy to Enhance Anti-tumor Effects of Nectin-4-MMAE**

Yichen Wang^1^, Yanyang Nan^2^, Chunguang Ma^1^, Xiaolin Lu^1^, Qian Wang^2^, Xiting Huang^2^, Wenjing Xue^2^, Jiajun Fan^2^, Dianwen Ju^2, #^, Dingwei Ye^1, #^, Xuyao Zhang^2, #^

^1^Deparatment of Urology, Fudan University Shanghai Cancer Center; Department of Oncology, Shanghai Medical College, Fudan University, Shanghai, 200032, China

^2^Department of Biological Medicines & Shanghai Engineering Research Center of Immunotherapeutic, Fudan University School of Pharmacy, Shanghai, 201203, China

**Running title**: Nectin-4-MMAE and Autophagy in bladder cancer

**^#^Corresponding author**

Dianwen Ju, [dianwenju@fudan.edu.cn](mailto:dianwenju@fudan.edu.cn)

Dingwei Ye, [dwyeli@163.com](mailto:dwyeli@163.com)

Xuyao Zhang, [xuyaozhang@fudan.edu.cn](mailto:xuyaozhang@fudan.edu.cn)

Figure S1


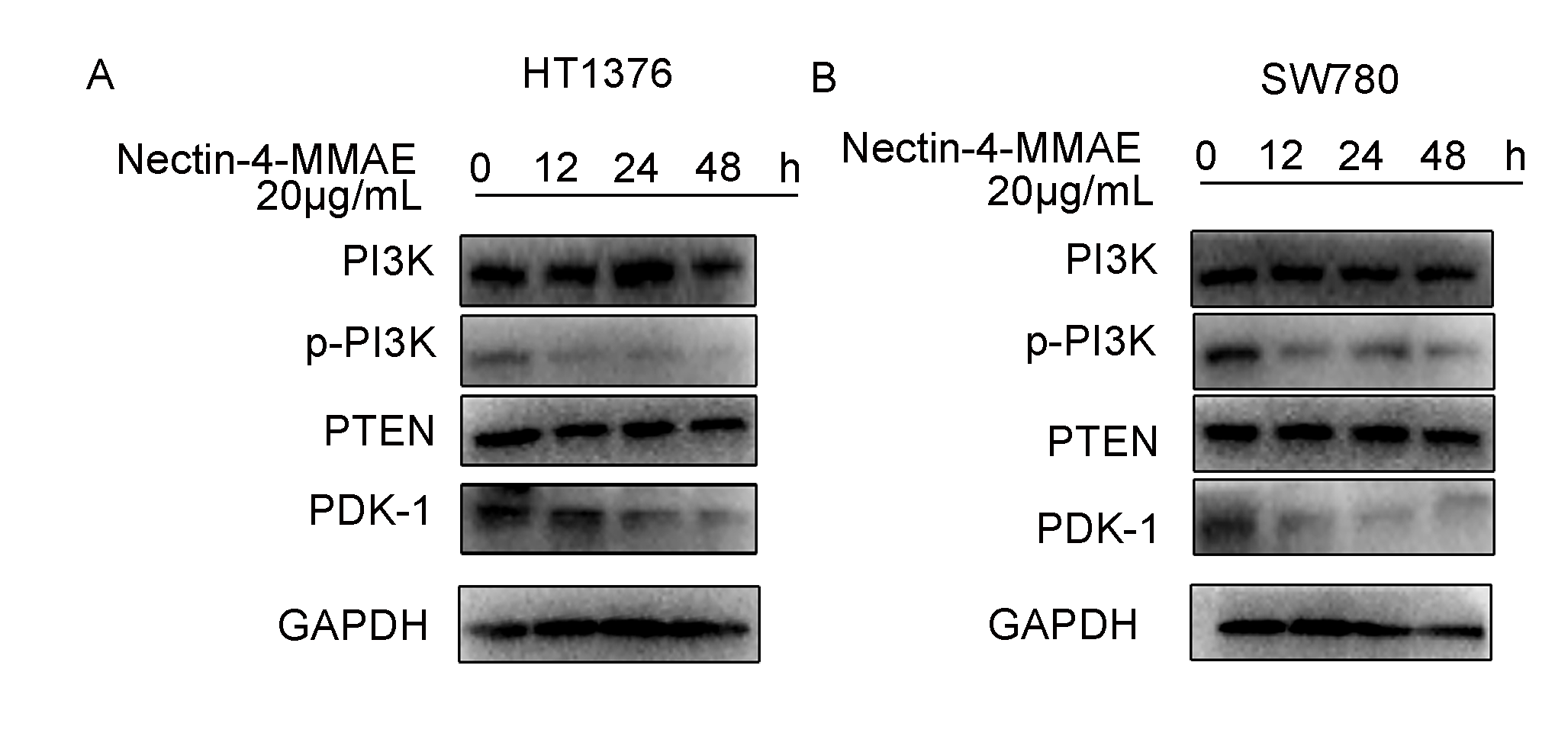


Figure S1 PI3K and PDK-1 were downregulated after Nectin-4-MMAE treatment. A, B. Western blot assay for Akt/mTOR upstream regulator PI3K (total PI3K and p-PI3K), PTEN, and PDK-1 showed that the phosphorylation level of PI3K and expression level of PDK-1 were both decreased after Nectin-4-MMAE treatment.
