# Supplementary material for: A potential strategy for bladder cancer treatment: inhibiting autophagy to enhance antitumor effects of Nectin-4-MMAE
Source: Cell Death Dis. 2024 Apr 25;15(4):293. doi: 10.1038/s41419-024-06665-y (PMC11045801; doi:10.1038/s41419-024-06665-y)

**A Potential Strategy for Bladder Cancer Treatment: Inhibiting Autophagy to Enhance Anti-tumor Effects of Nectin-4-MMAE**

Yichen Wang^1^, Yanyang Nan^2^, Chunguang Ma^1^, Xiaolin Lu^1^, Qian Wang^2^, Xiting Huang^2^, Wenjing Xue^2^, Jiajun Fan^2^, Dianwen Ju^2, #^, Dingwei Ye^1, #^, Xuyao Zhang^2, #^

^1^Deparatment of Urology, Fudan University Shanghai Cancer Center; Department of Oncology, Shanghai Medical College, Fudan University, Shanghai, 200032, China

^2^Department of Biological Medicines & Shanghai Engineering Research Center of Immunotherapeutic, Fudan University School of Pharmacy, Shanghai, 201203, China

**Running title**: Nectin-4-MMAE and Autophagy in bladder cancer

**^#^Corresponding author**

Dianwen Ju, [dianwenju@fudan.edu.cn](mailto:dianwenju@fudan.edu.cn)

Dingwei Ye, [dwyeli@163.com](mailto:dwyeli@163.com)

Xuyao Zhang, [xuyaozhang@fudan.edu.cn](mailto:xuyaozhang@fudan.edu.cn)

Supplemental material

Original data (western blot)


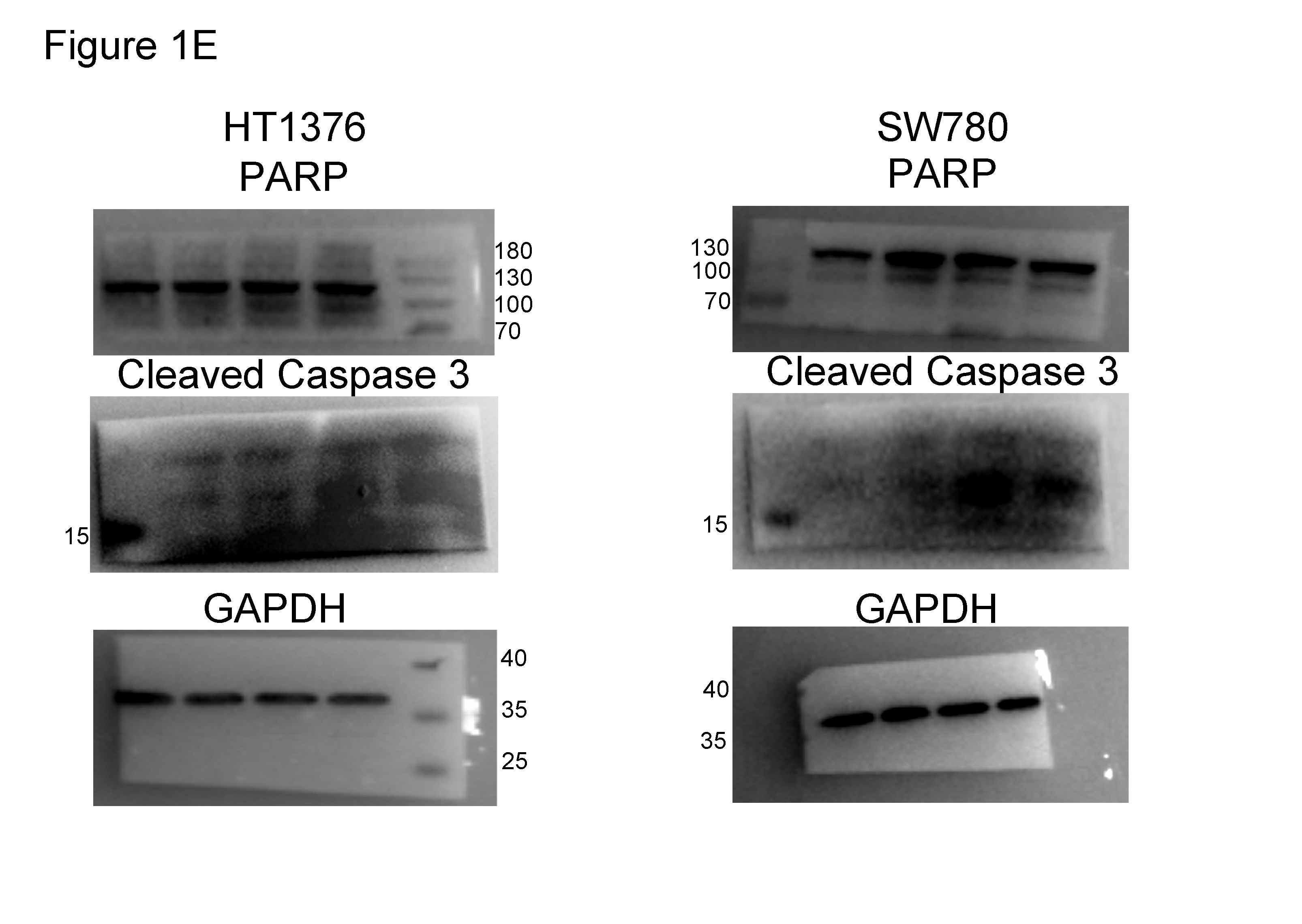


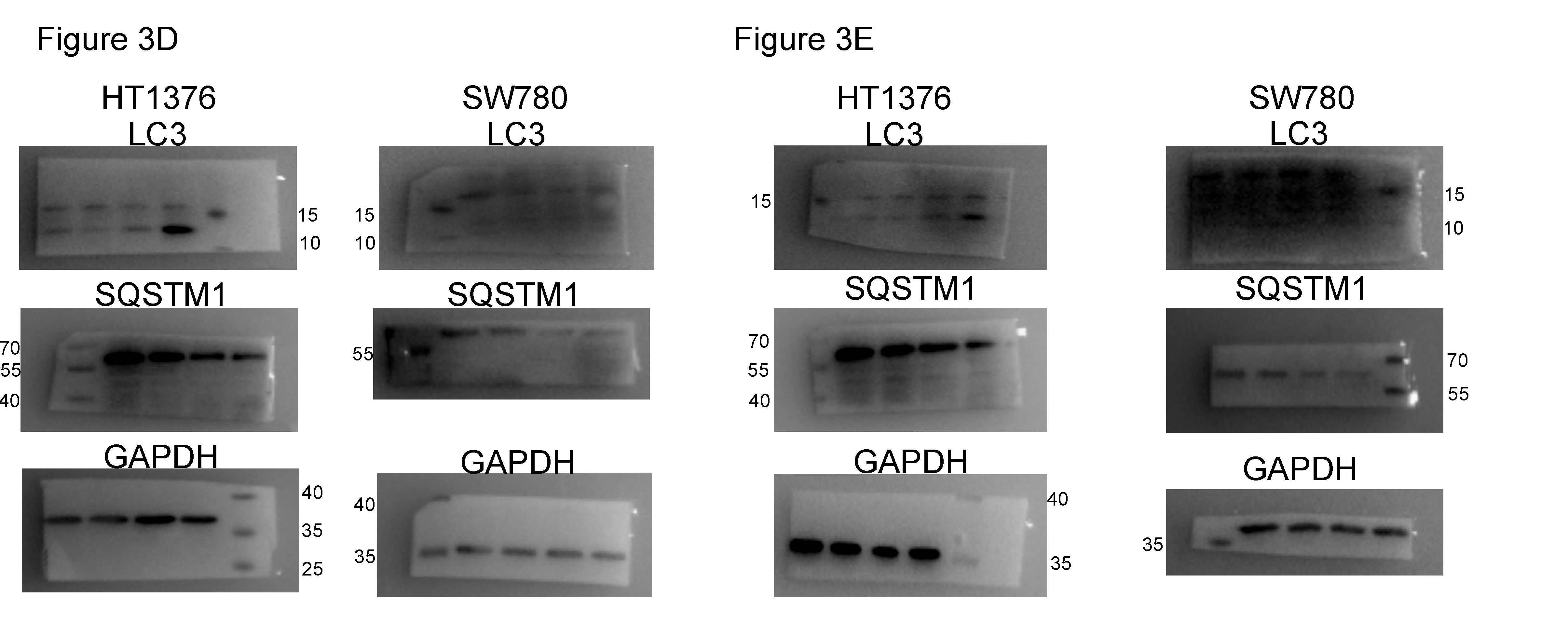


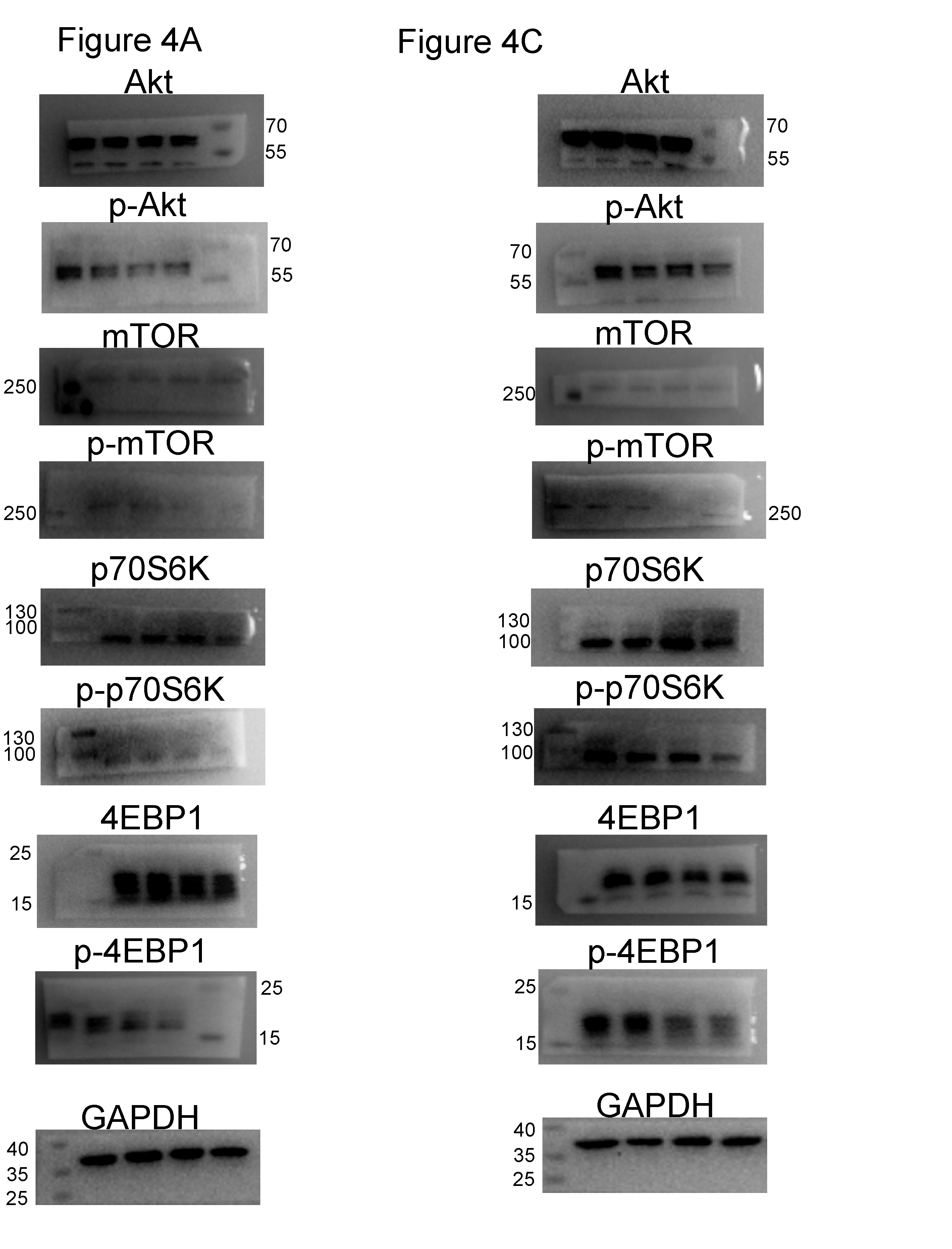


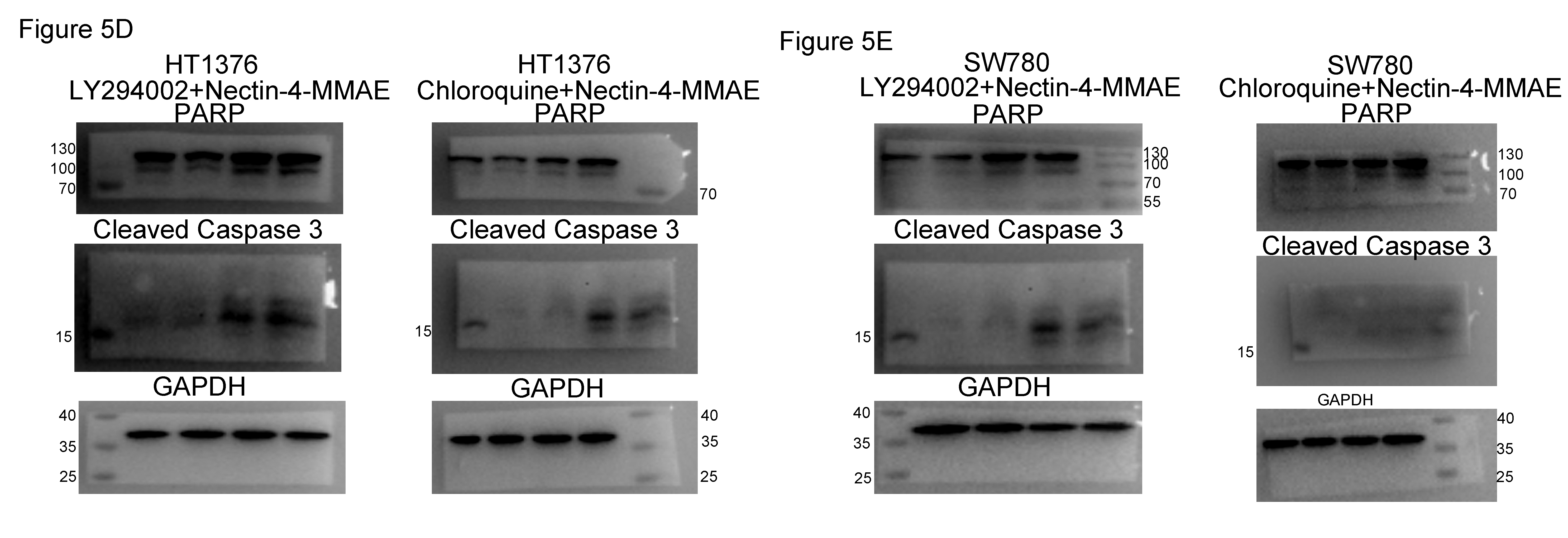


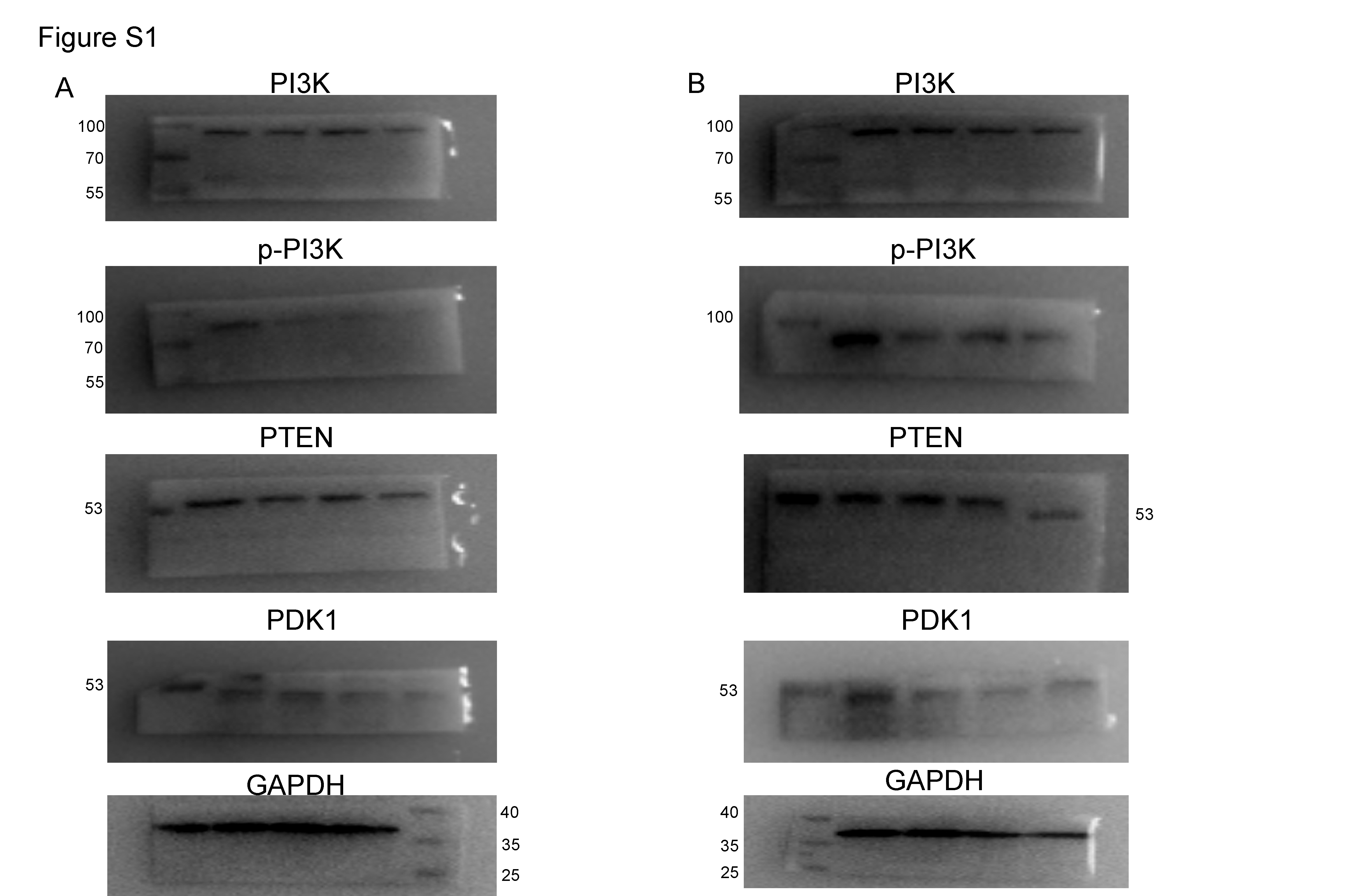

Supplement: Supplementary file 2 — Supplemental material (western blot) [file 41419_2024_6665_MOESM2_ESM.docx]
